# Supplementary material for: The structural pathology for hypophosphatasia caused by malfunctional tissue non-specific alkaline phosphatase
Source: Nat Commun. 2023 Jul 8;14:4048. doi: 10.1038/s41467-023-39833-3 (PMC10329691; doi:10.1038/s41467-023-39833-3)
Supplement: Supplementary file 1 — Supplementary Information [file 41467_2023_39833_MOESM1_ESM.pdf]

Supplementary information for

The structural pathology for hypophosphatasia caused by malfunctional tissue non-specific alkaline phosphatase

Yating Yu[1,2†], Kewei Rong[1†], Deqiang Yao[3†], Qing Zhang[1,2], Xiankun Cao[1], Bing Rao[1,2], Ying Xia[2], Yi Lu[2], Yafeng Shen[2], Ying Yao[4], Hongtao Xu[4], Peixiang Ma[1*], Yu Cao[1,2*], An Qin[1*]

Supplementary items:

Supplementary Figures 1-17

Supplementary Tables 1-3

Supplementary Data 1

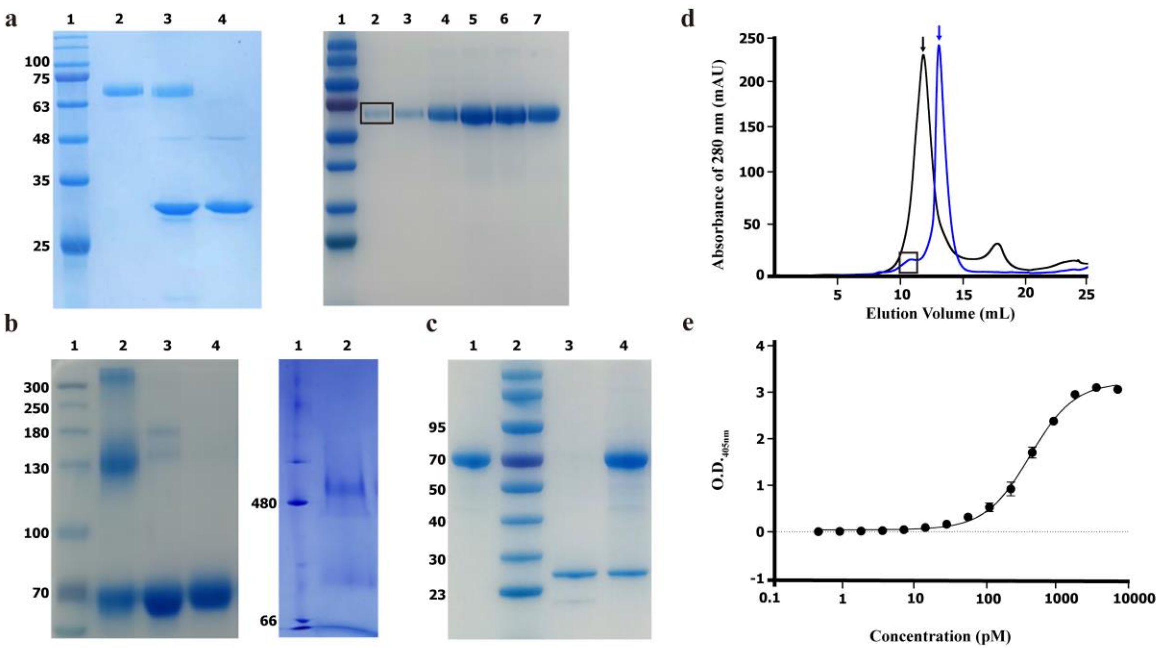

Supplementary Figure 1. The purification of the hTNAP$^{18\text{-}500}$ and the hTNAP$^{18\text{-}500}$-scFv complex. a: The SDS-PAGE analysis on the protein purification of hTNAP$^{18\text{-}500}$. Left: lane 1 - molecular weight standard (kD); lane 2 - the eluate from the affinity resin; lane 3 - the eluate from the affinity resin treated with TEV protease; and lane 4 - TEV protease. Right: lane 1 - molecular weight standard (kD). from lane 2 to lane 7 - the peak fractions (indicated with the blue arrow and black fame in d) eluted from size exclusion chromatography. **b:** The cross-linking and blue native PAGE detection of the hTNAP$^{18\text{-}500}$ oligomeric states. Left: lane 1 - molecular weight standard (kD); lane 2 to lane 4 – hTNAP$^{18\text{-}500}$ protein treated with crosslinking reagents GA, DMP, and Sulfo-LC-SPDP. Right: The native PAGE analysis results. lane 1 - molecular weight standard (kD); lane 2 - hTNAP$^{18\text{-}500}$ protein. **c:** The SDS-PAGE analysis on the protein purification of hTNAP$^{18\text{-}500}$-scFv complex. lane 1 - TNAP$^{18\text{-}500}$ protein purified by SEC-FPLC; lane 2 - molecular weight standard (kD); lane 3 - scFv protein purified by SEC-FPLC; from lane 4 - the peak (indicated with the black arrow in d) fractions of the TNAP$^{18\text{-}500}$-scFv complex eluted from size exclusion chromatography. **d:** The size exclusion chromatography profiles of the hTNAP$^{18\text{-}500}$ (blue) and the hTNAP$^{18\text{-}500}$-scFv complex (black). **e:** The binding affinity for scFv-Fc (JTALP001) antibody with TNAP was determined by ELISA. TNAP$^{18\text{-}500}$ protein, purified from insect cells, was coated on the ELISA plates and probed with increasing concentrations of antibodies. The K_D was 401 ± 15 pM. optical density (OD). n = 3 biologically independent samples. All data in this figure are represented as mean ± SD. All experiments were repeated three times independently with similar results. Source data are provided as a Source Data file.

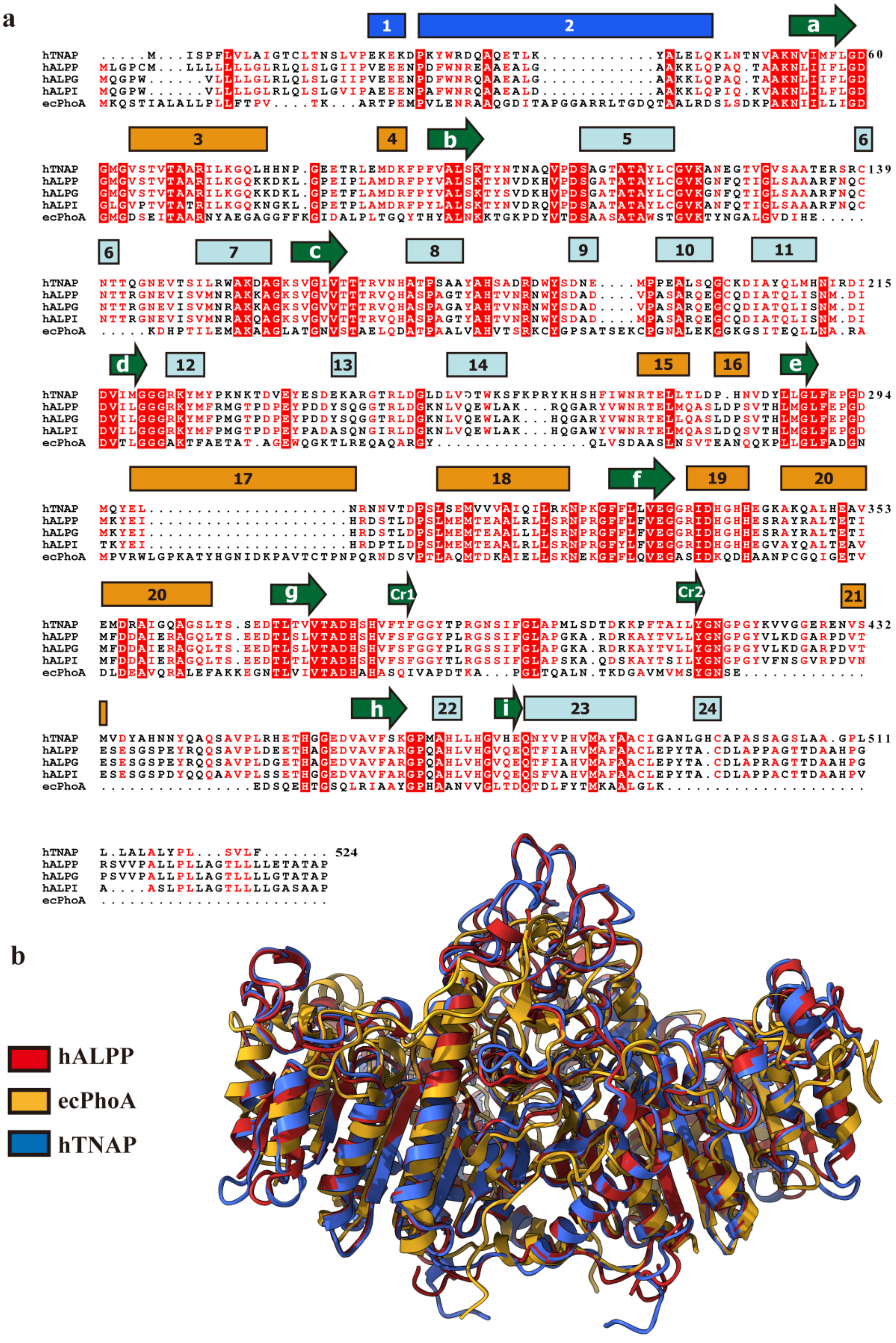
a
1
2
a
3
4
b
5
6
7
c
8
9
10
11
d
12
13
14
15
16
e
17
18
f
19
20
g
Cr1
Cr2
21
h
22
i
23
24
hTNAP
hALPP
hALPG
hALPI
ecPhoA
60
139
215
294
353
432
511
524
b
hALPP
ecPhoA
hTNAP

Supplementary Figure 2. The sequence and structural comparison among alkaline phosphatases. a: The sequence alignment among alkaline phosphatases from *E. coli*, human TNAP, human ALPG and human ALPI. The protein sequences of *E. coli* alkaline phosphatase (ecPhoA, UniProtKB - P00634), placental alkaline phosphatase from *Homo sapiens* (hALPP, UniProtKB - P05187), tissue non-specific alkaline phosphatase from *Homo sapiens* (hTNAP, UniProtKB - P05186), intestinal-type alkaline phosphatase from Homo sapiens (hALPI, UniProtKB - P09923), and alkaline phosphatase of germ cell type from *Homo sapiens* (hALPG, UniProtKB - P10696) were aligned with the secondary structural elements of hTNAP marked above the alignment. Residues are colored based on their conservation using the ESPript server. **b:** A structural superposition between hTNAP and hPLAP structures focusing on the calcium-binding site (left) and active site (right). The TNAP and PLAP (PDB ID 1EW2) structures were shown as cartoon models, with ions being shown as spheres and key residues in ion binding and enzymatic activity being shown as a stick model.

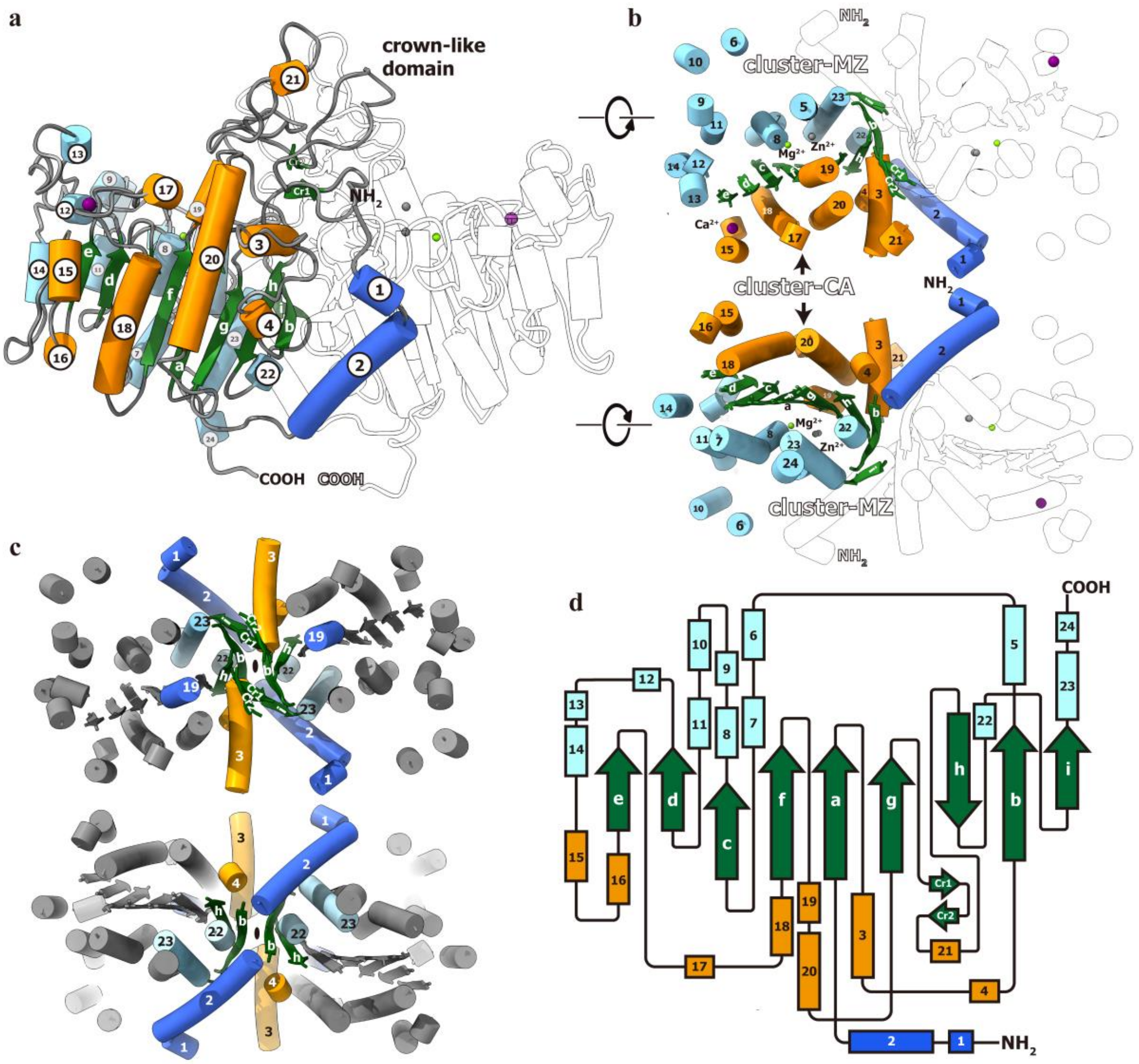

Supplementary Figure 3. The secondary structures organization of hTNAP$^{18\text{-}500}$. a: The dimeric hTNAP$^{18\text{-}500}$ shown as cartoon model. One protomer of the TNAP dimer was colored in by domains where the N-terminal helices (H1 and 2) were colored in blue, β strands (a-i, Cr1, and Cr2) in green, helices within cluster-CA (H3, 4, and 15-21) in orange and cluster-MZ (H5-14, and 22-24) in cyan. The other protomer was shown as black lines, and the metal ions in the dimer were shown as balls colored in purple (Ca^{2+}), green (Mg^{2+}), and gray (Zn^{2+}). **b:** The cartoon model of the dimeric hTNAP$^{18\text{-}500}$ viewed from the top and bottom of a. **c:** The dimeric interface of the hTNAP$^{18\text{-}500}$. The helices and β strands involved with dimerization were colored as the color palette shown in a and b, while others colored in gray. **d:** Cartoon representation of the organization of the secondary structures in hTNAP$^{18\text{-}500}$.

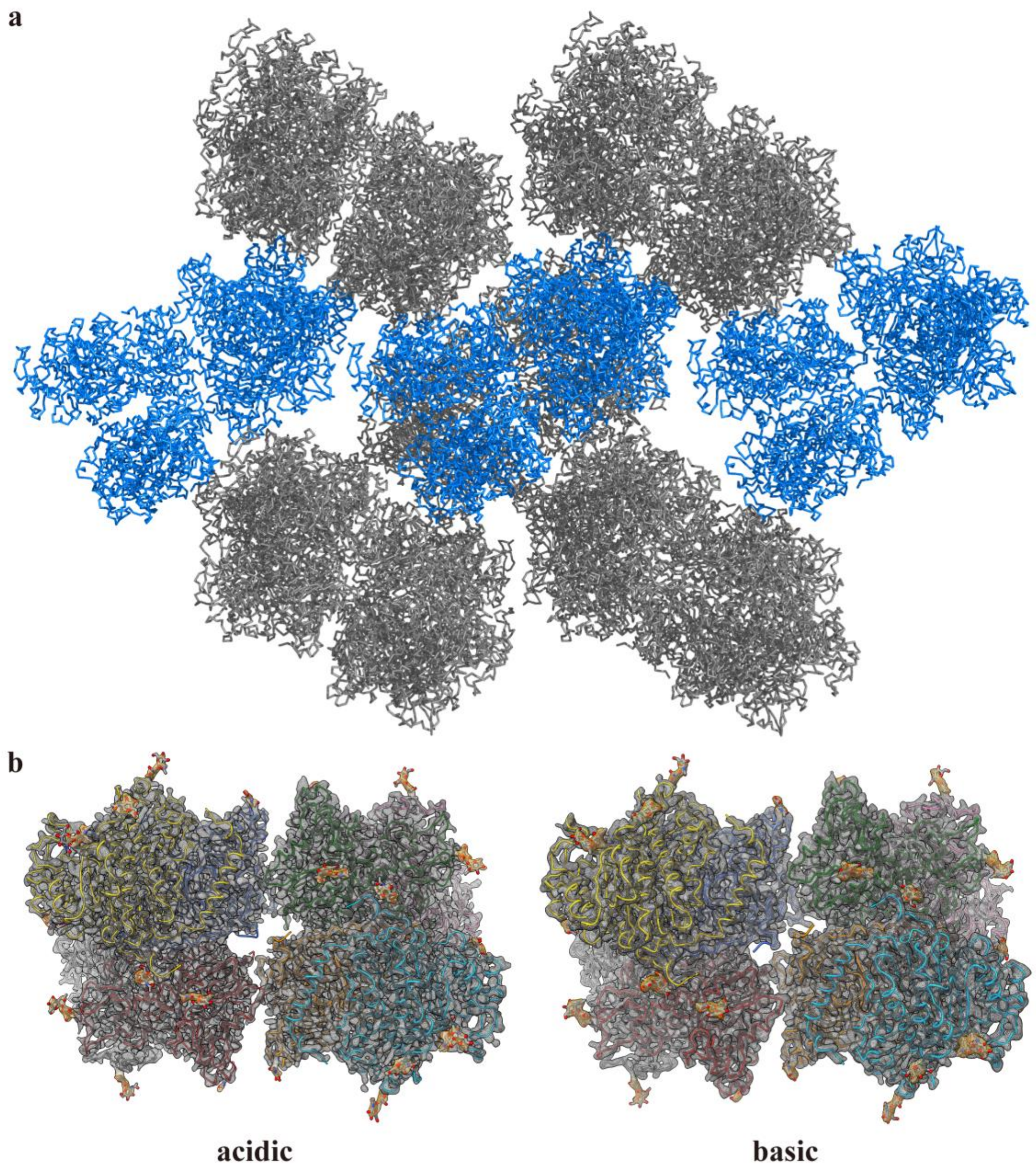

Supplementary Figure 4. The X-ray Crystallography of hTNAP$^{18\text{-}500}$. a: Crystal packing of hTNAP$^{18\text{-}500}$. **b:** The molecular model of the crystal structure of hTNAP$^{18\text{-}500}$ octamers and the corresponding electron density.

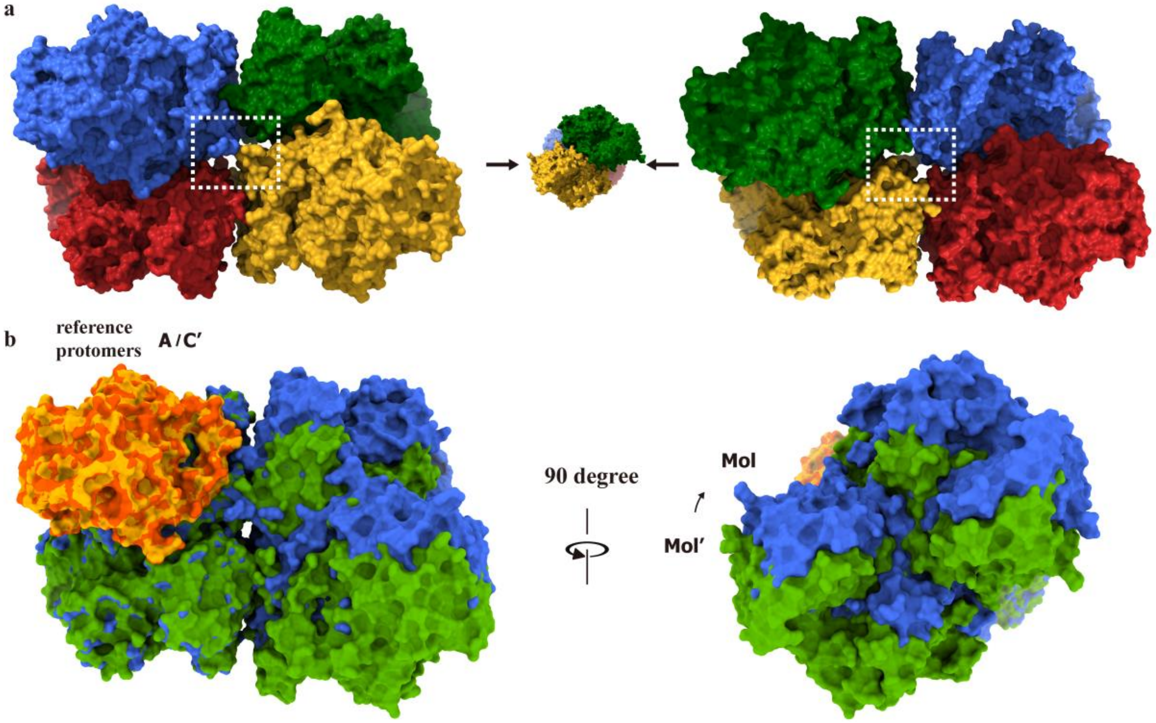

Supplementary Figure 5. The asymmetry of the octameric structure of hTNAP$^{18\text{-}500}$. a: The hTNAP$^{18\text{-}500}$ octamer was shown as surface model and viewed from two opposite angles. The dotted frame indicates the different central structures on the two sides. **b:** The self-superposition of the hTNAP$^{18\text{-}500}$ octamer. The octameric structure of the hTNAP was duplicated and superposed using the protomer A of the original structure (blue) to align with the protomer C (marked as C') of the structure copy (green).

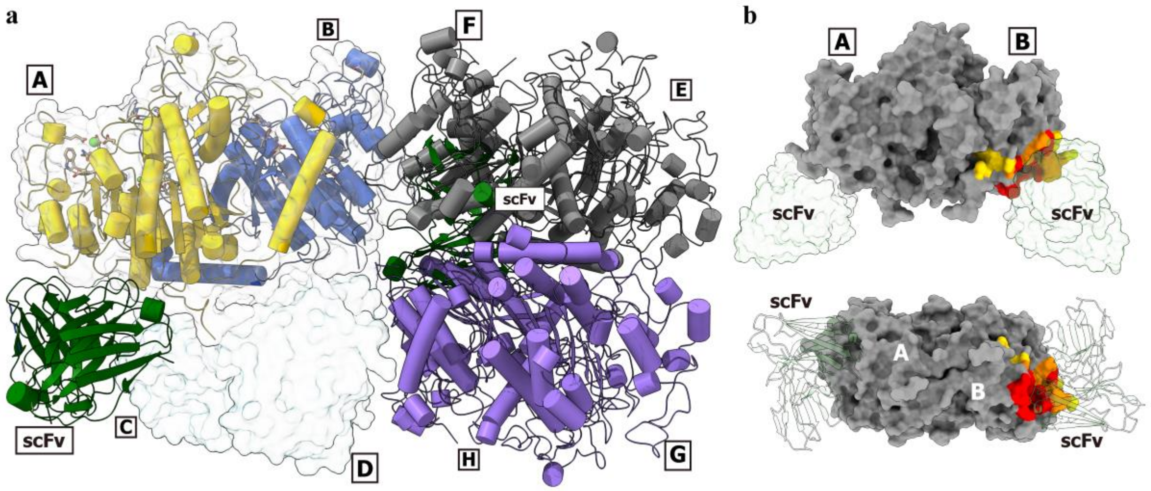

Supplementary Figure 6. The spatial overlapping of the octameric interface and the antibody epitope. a: The superposition between the hTNAP$^{18\text{-}500}$ octamer and hTNAP$^{18\text{-}500}$-scFv complex using the protomer A and B as reference. The protomer A-D in octamer were shown as the surface model, and protomer E-H, as well as TNAP-scFv, were shown as cartoon models. The TNAP dimer bound with scFv was superposed with the protomer A and B, with the scFv (green model) binding with protomer B overlapping with the protomer F and H of the octamer. **b:** The surface model of the hTNAP$^{18\text{-}500}$-scFv. The TNAP dimer was colored in gray, and the scFv was colored in translucent green. The octameric interface was colored in yellow on the surface model of protomer B and the epitope was colored in red, with the overlapping region colored in orange.

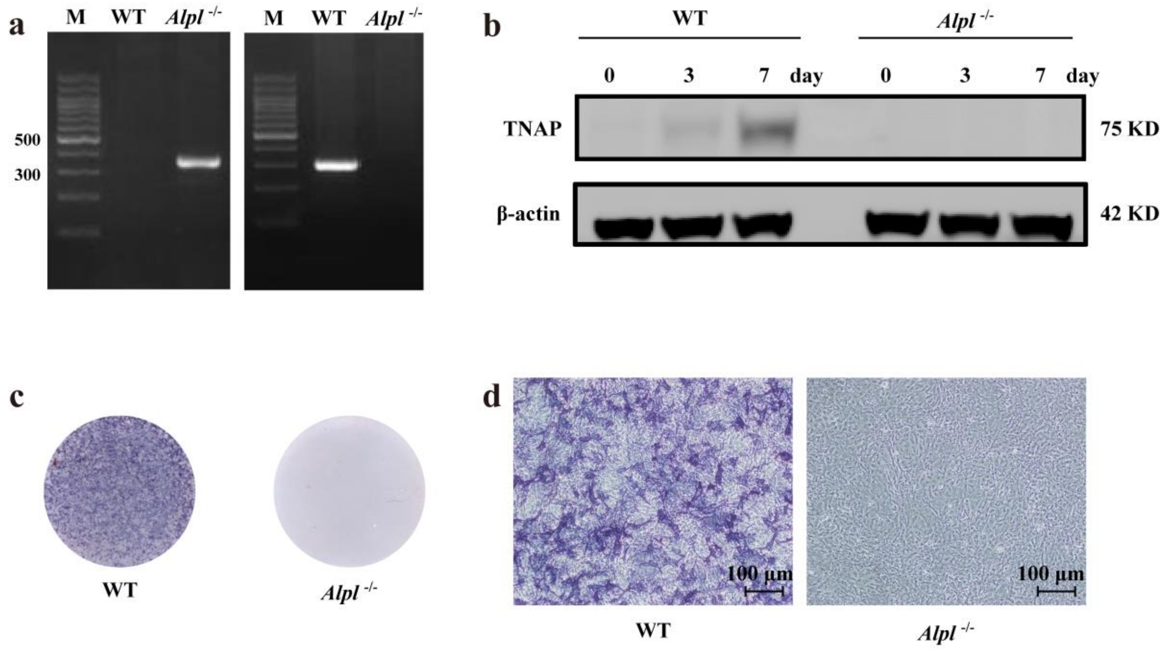

Supplementary Figure 7. Verification of the knockout efficiency in Alpl$^{-/-}$ mice. a: Genotyping analysis from tail-tip DNA of Alpl$^{-/-}$ mice or WT. M: molecular weight standard (bp). WT: PCR-i reaction without product; PCR-ii reaction obtains a single WT band. Alpl$^{-/-}$: PCR-i reaction obtains a single KO band; PCR-ii reaction without product. **b:** Western blotting of TNAP protein at 0, 3, and 7 days of pre-osteoblasts during osteogenic differentiation. **c, d:** ALP staining of calvaria-derived pre-osteoblasts after differentiation for 7 days (left). Amplified pictures showing details (right). All experiments were repeated three times independently with similar results. Source data are provided as a Source Data file.

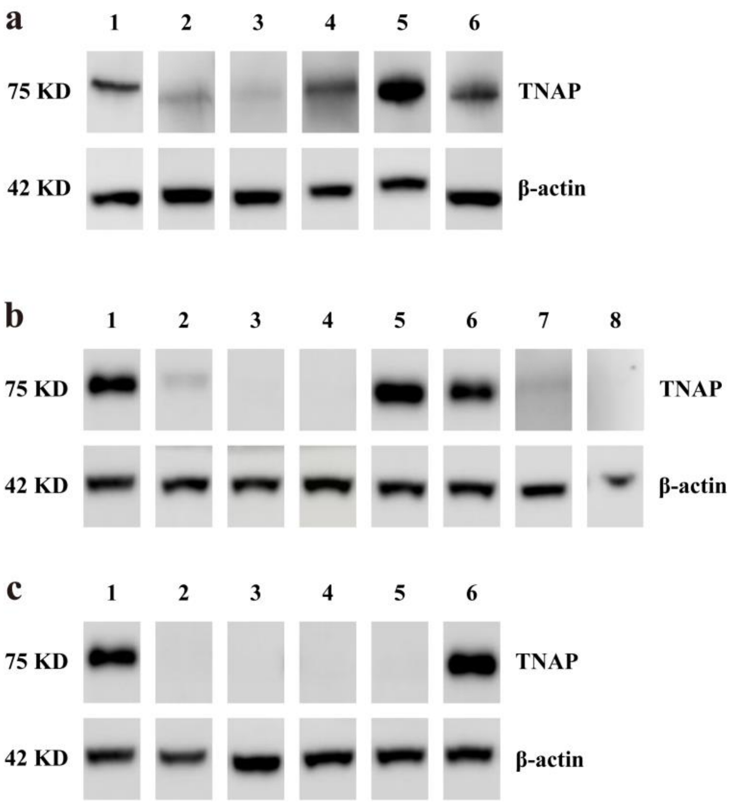

Supplementary Figure 8. a: The expression levels of TNAP with dimeric mutations. 1-6: immunoblotting results of HEK293T cell overexpressing hTNAP wild-type, hTNAP^{G420S}, hTNAP^{R71H}, hTNAP^{R391H}, hTNAP^{R450C}, and hTNAP^{G420A}. **b:** The expression levels of TNAP with interface S mutations. 1-8: immunoblotting results of HEK293T cell overexpressing hTNAP wild-type, hTNAP^{D156Y}, hTNAP^{G491R}, hTNAP^{C497S}, hTNAP^{K264R}, hTNAP^{R152H}, hTNAP^{I490F}, and hTNAP^{C489S}. **c:** The expression levels of TNAP with interface L mutations. 1-6: immunoblotting results of HEK293T cell overexpressing hTNAP wild-type, hTNAP^{Y28D}, hTNAP^{E311K}, hTNAP^{T366N}, hTNAP$^{\Delta S368}$, and hTNAP^{S368A}. All experiments were repeated three times independently with similar results. Source data are provided as a Source Data file.

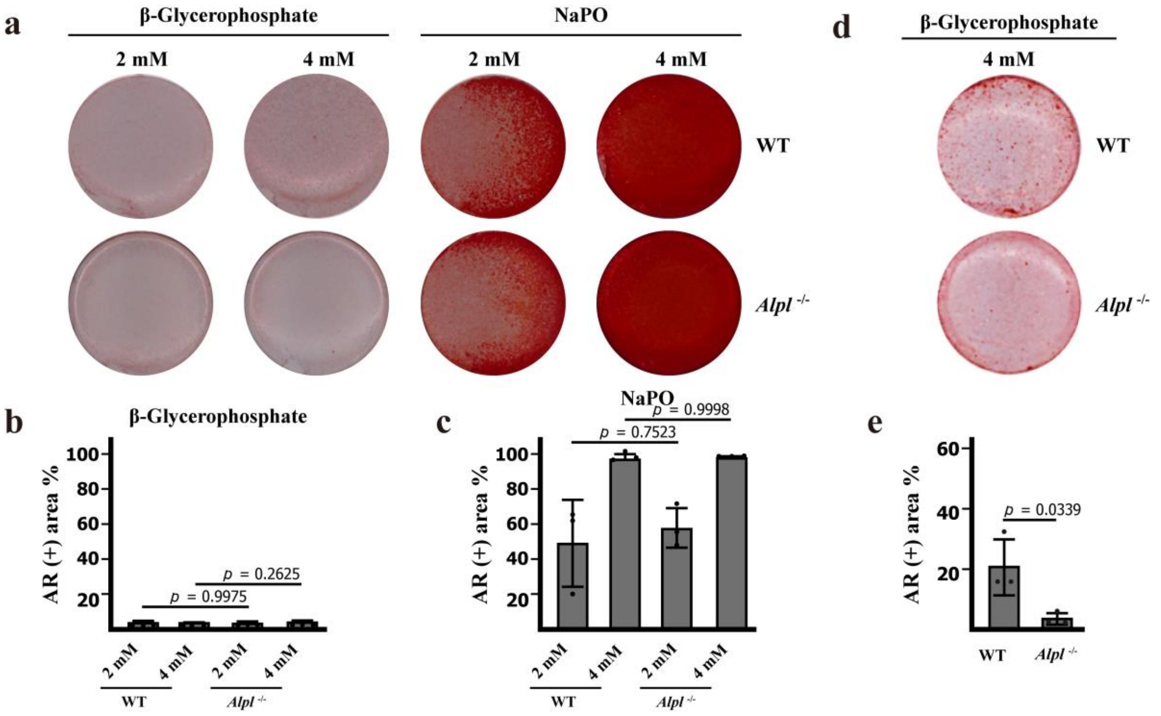

Supplementary Figure 9. Alizarin red staining of Wild-type and Alpl$^{-/-}$ mice-derived pre-osteoblasts. a-c: Alizarin red staining of Wild-type and Alpl$^{-/-}$ mice-derived pre-osteoblasts on day 14. 2mM and 4mM NaPO or β-glycerophosphate were used(top). Quantification of the percentage of Alizarin red (+) area (bottom). n = 3 biologically independent samples. **d-e:** Alizarin red staining of Wild-type and Alpl$^{-/-}$ mice-derived pre-osteoblasts on day 21. 4mM β-glycerophosphate were used (top). Quantification of the percentage of Alizarin red (+) area (bottom). n = 3 biologically independent samples. All data in this figure are represented as mean ± SD. One-way ANOVA with Tukey's multiple comparisons test for (b, c) and unpaired two-tailed Student's t-test for (e). All experiments were repeated three times independently with similar results. Source data are provided as a Source Data file.

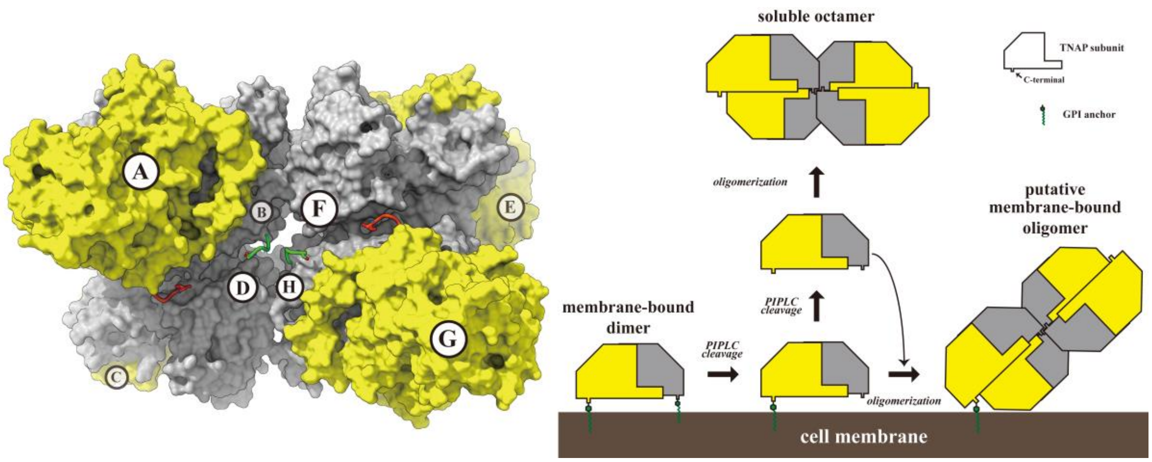

Supplementary Figure 10. The octameric TNAP and GPI-anchors: Left: the surface model of TNAP octamer showing its GPI-anchoring sites. The eight TNAP subunits were shown as surface model and colored in yellow (protomers A, D, E, and G) and gray (protomers B, D, F, and H). The C-terminal serine residues for linkage to the GPI moieties were shown as stick model and colored in red (protomers A and G) and green (protomers B and H). **Right:** a proposed working model for TNAP octamerization. This procedure started from the dimeric membrane-bound form with both protomers bearing GPI. The enzymatic cleavage will remove the GPI anchor and release the dimeric TNAP into the extracellular environment, thereby forming a soluble octamer. Alternatively, a dimeric TNAP with only one GPI anchor could recruit soluble dimeric TNAP to form a putative membrane-bound octamer.

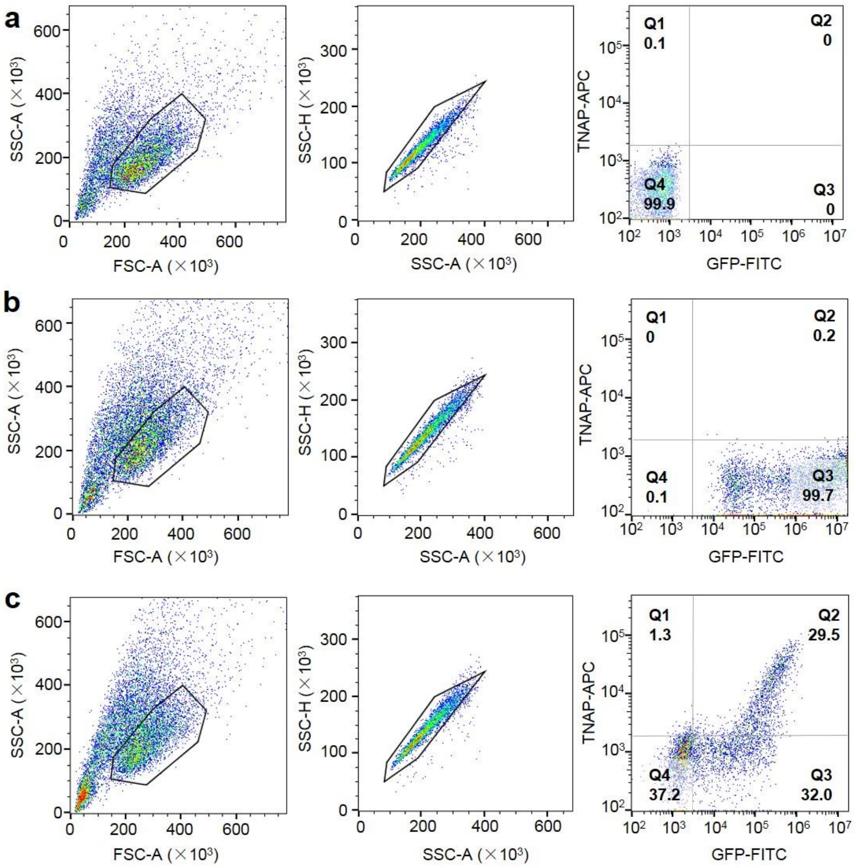

d

	Q1	Q2	Q3	Q4
HEK293T-1	0.1	0.0	0.0	99.9
HEK293T-2	0.0	0.0	0.0	100.0
HEK293T-3	0.0	0.0	0.0	100.0
HEK293T-GFP-1	0.0	0.2	99.7	0.1
HEK293T-GFP-2	0.0	0.0	99.9	0.1
HEK293T-GFP-3	0.0	0.1	99.9	0.0
HEK293T-GFP-hTNAP-1	1.3	29.5	32.0	37.2
HEK293T-GFP-hTNAP-2	1.3	29.0	33.3	36.4
HEK293T-GFP-hTNAP-3	1.2	26.8	32.5	39.5

Supplementary Figure 11. The binding of JTALP001 antibody to surface-expressed TNAP. a. HEK293T cells were used to set the gates. Forward Scatter (FSC-A) vs Side Scatter (SSC-A) plot was used to remove most of the debris, air bubbles, and laser noise in the first step. The doublets and multiplets were excluded using FSC-H vs SSC-A plots. The hTNAP antibody recognized hTNAP transfected cells were in Q2. **b & c.** HEK293T cells transfected with EGFP (b) and HEK293T cells transiently transfected with the plasmids encoding the full-length hTNAP in fusion with P2A-EGFP (c) were incubated with purified JTALP001 antibody and stained with Alexa 633 labeled anti-human secondary antibody, then analyzed by FACS. All experiments were repeated three times independently and the population were listed in **d**.

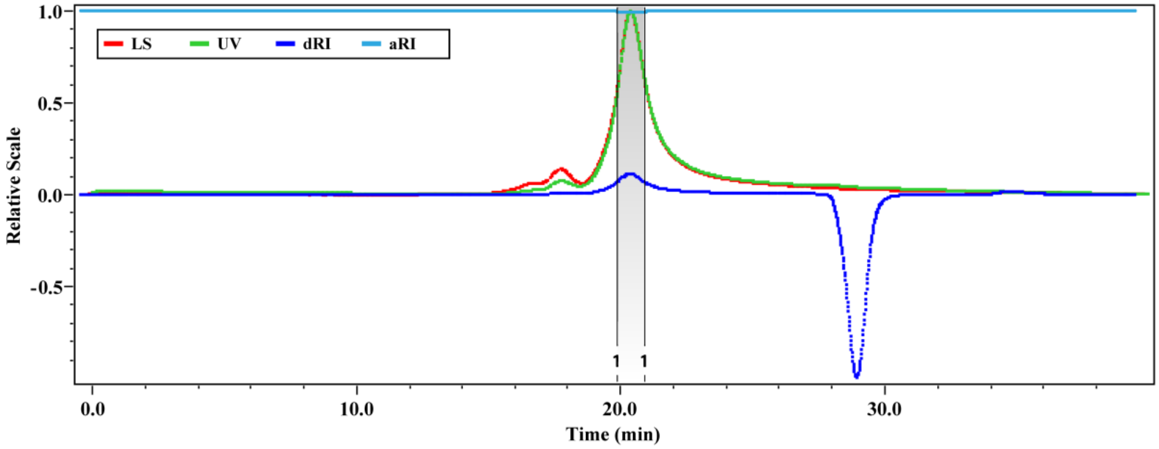

Supplementary Figure 12. The static light scattering (SLS) of hTNAP. The peak results showed the molar mass moments (g/mol) of hTNAP. The Mn was 1.226×10^5 (±0.326%) and Mw was 1.227×10^5 (±0.327%). All experiments were repeated three times independently with similar results. Source data are provided as a Source Data file.

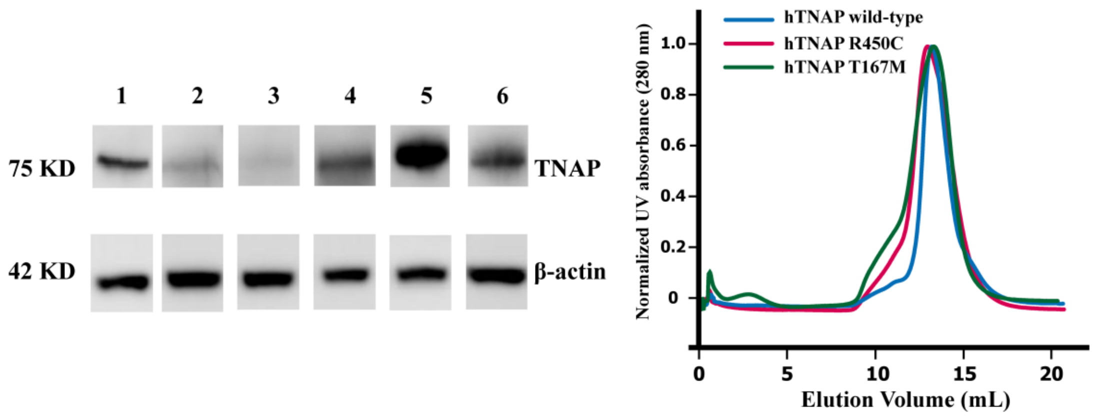

Supplementary Figure 13. The effects of mutations at dimeric interface on the expression level of and SEC profile TNAP. Left: The expression levels of TNAP with dimeric mutations. 1-6: immunoblotting results of HEK293T cell overexpressing hTNAP wild-type, G420S, R71H, R391H, R450C, and G420A. **Right:** The SEC FPLC profile for $hTNAP^{R450C}$ (red), $hTNAP^{T167M}$ (green), and hTNAP wild-type (blue). All experiments were repeated three times independently with similar results. Source data are provided as a Source Data file.

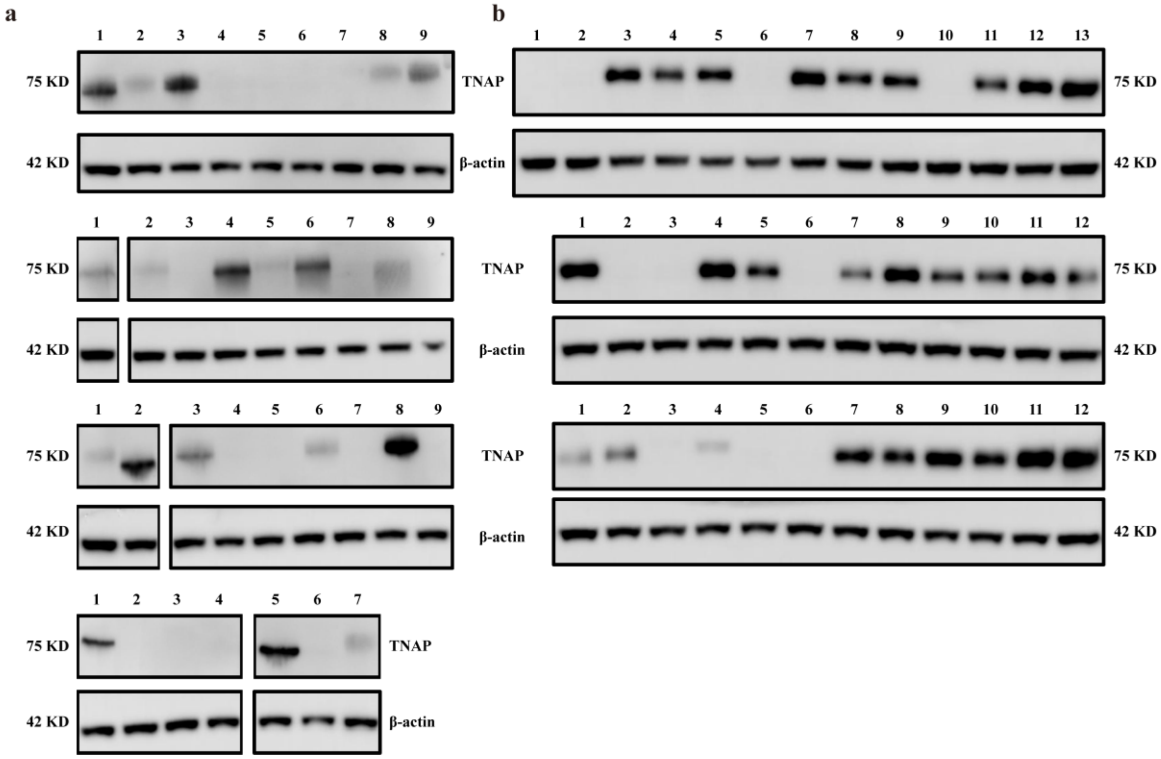

Supplementary Figure 14. Western blot of the wild-type and mutations of hTNAP$^{18\text{-}500}$ proteins. a: First two layers: lane 1 to 9 - G420A, D294A, N417S, C497S, C201Y, D306V, G334D, H171R, R184W; Second two layers: lane 1 to 9 - G420S, R71H, N170D, T167M, I490F, R391H, G491R, N430S, C489S; Third two layers: lane 1 to 9 - Y28D, P499N, K264R, E291K, E235G, H171Y, T366N, R450C, D156Y; Fourth two layers: lane 1 to 7 -wild-type, Reagent control, Vector control, Blank, R152H, S368del, A116T. **b:** First two layers: lane 1 to 13 - Blank, Reagent control, wild-type, K22A, D25A, Y28D, D31A, E35A, K38A, E311K, K45A, Q318A, R321A; Second two layers: lane 1 to 12 - S364A, T366N, S368del, S368A, E369A, D370A, D370E, R357L, H78A, H79A, N80A, G420A; Third two layers: lane 1 to 12 - G420S, R213A, D214A, D156Y, G491R, C497S, K325A, Y263H, K264R, R152H, R450C, T167M. All experiments were repeated three times independently with similar results. Source data are provided as a Source Data file.

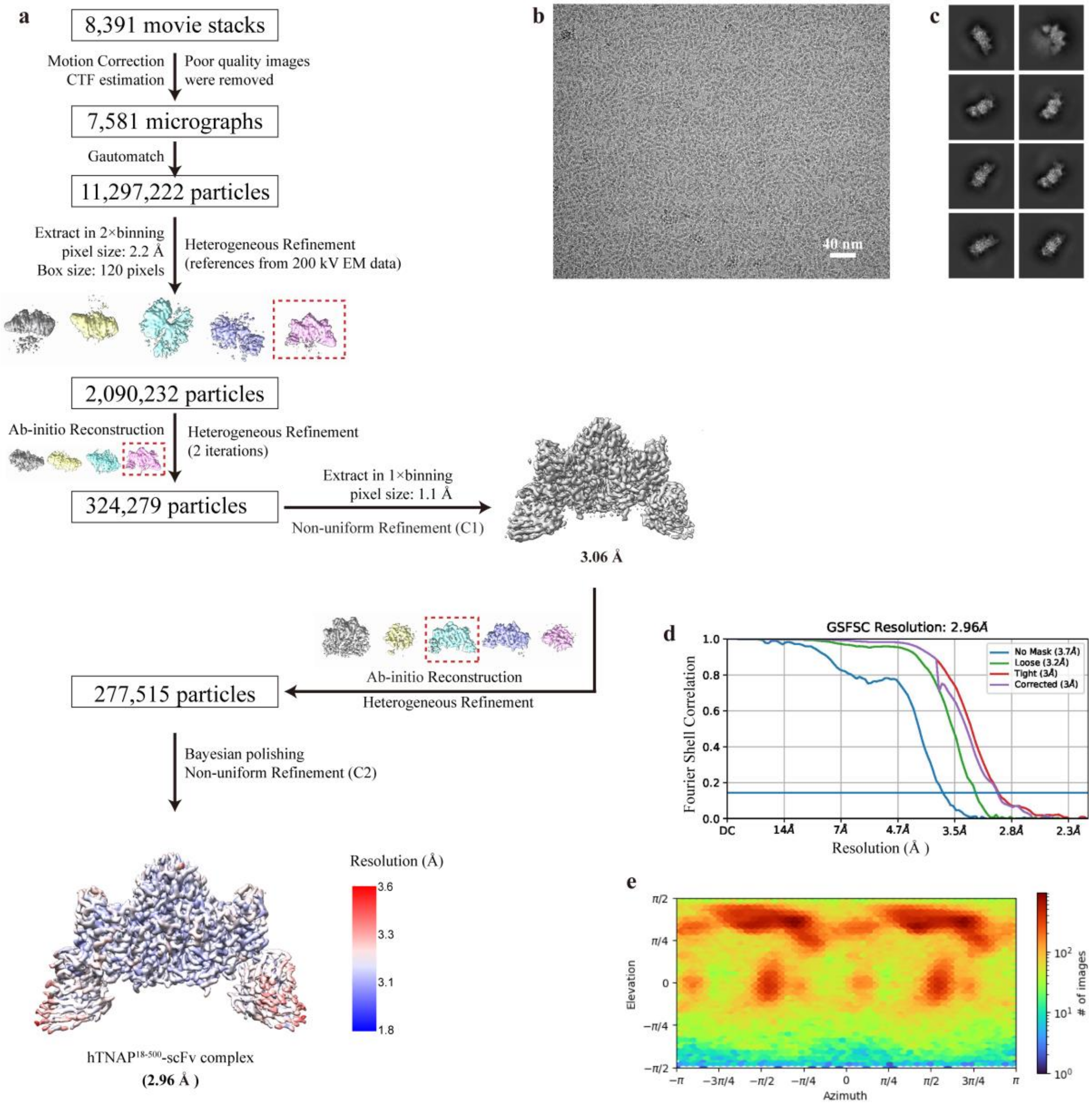

Supplementary Fig. 15. The cryo-EM analysis of hTNAP$^{18\text{-}500}$-scFv complex. a. The flow chart of cryo-EM data processing on hTNAP$^{18\text{-}500}$-scFv complex. Local-resolution map shown in red-white-blue. **b.** A representative cryo-EM micrograph of hTNAP$^{18\text{-}500}$-scFv complex. Most of the micrographs were similar with high quality. **c.** A representative 2D class averages. **d.** The gold-standard Fourier shell correlation (FSC) curve for the final cryo-EM map of hTNAP$^{18\text{-}500}$-scFv complex, generated by cryoSPARC with non-uniform refinement. **e.** Orientation distribution of particles for the final map reconstruction.

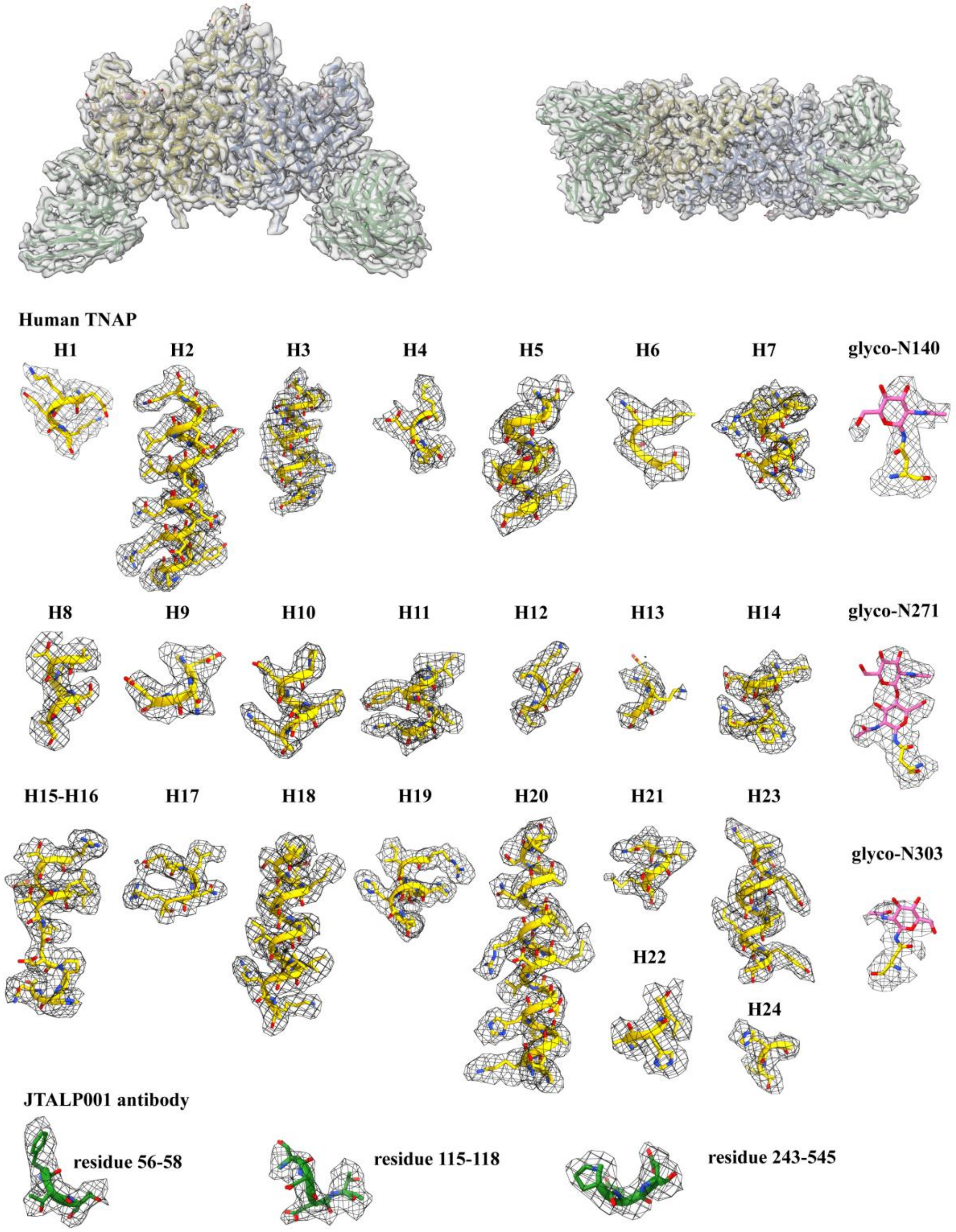

Supplementary Fig. 16. The EM density and the model-fitting for the key secondary structures of hTNAP$^{18-500}$-scFv complex. The helices H1-24 of hTNAP$^{18-500}$ (in yellow), glycosylated asparagine residues (in pink), and three short helices in scFv JTALP001 (in green) were shown combined with their corresponding cryo-EM density map. All the density maps are shown at the same contour level.

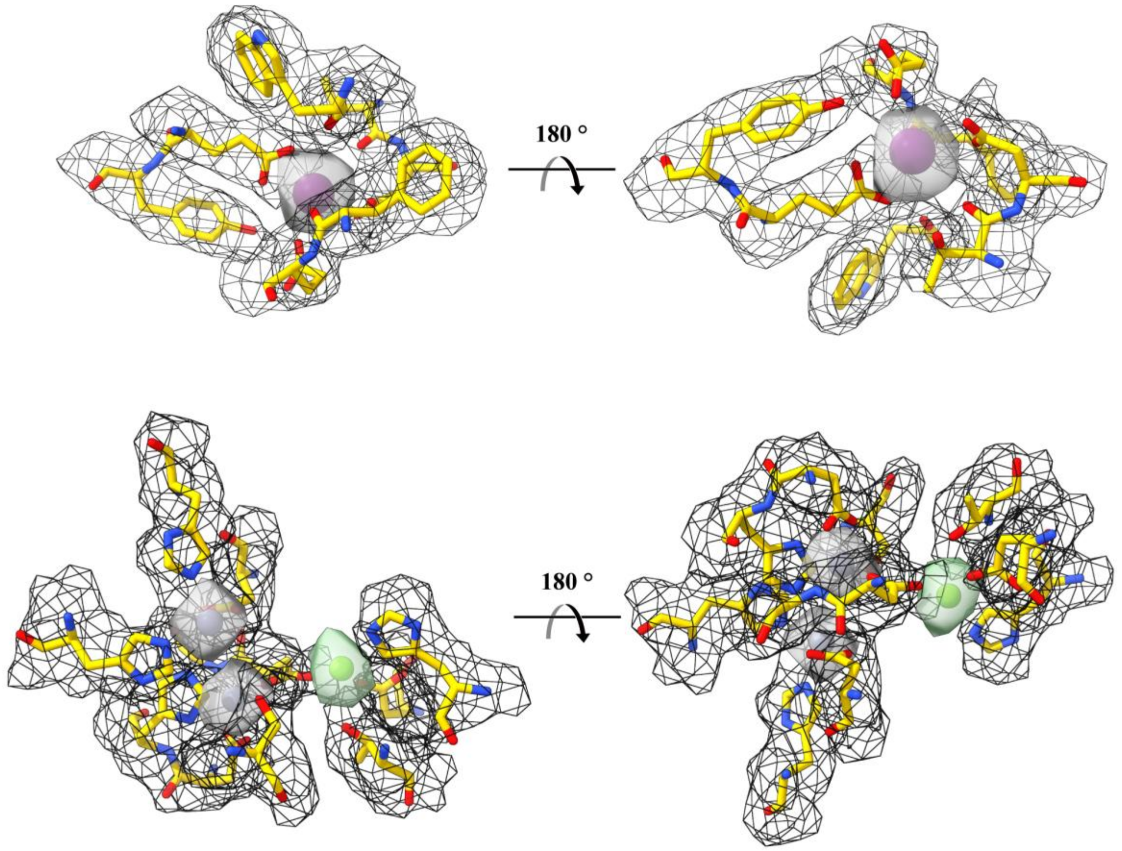

Supplementary Fig. 17. The EM density and the model-fitting for the active site and calcium-binding site of hTNAP$^{18\text{-}500}$-scFv complex. The residues and ions in the calcium binding site (upper) and active site (lower) were shown combined with their corresponding cryo-EM density map and viewed in two opposite angles. The calcium, magnesium and two zinc ions were shown as spheres in purple, green, and gray, respectively. All the density maps are shown at the same contour level.

Supplementary Table 1. The data collection and refinement statistics for hTNAP$^{18\text{-}500}$ crystal structure.

	Human TNAP$^{18\text{-}500}$, acidic pH (PDB ID 7YIW)	Human TNAP$^{18\text{-}500}$, basic pH (PDB ID 7YIV)
**Data collection**		
Space group	P $2_1 2_1 2_1$	P $2_1 2_1 2_1$
Cell dimensions		
a, b, c (Å)	159.36, 166.53, 181.60	158.62, 167.35, 188.78
α, β, γ (°)	90, 90, 90	90, 90, 90
Resolution (Å)	47.36 - 2.89	25.05 - 3.18
R_{merge}	0.2115	0.225
$I / \sigma I$	12.79 (2.64)	13.58 (2.28)
Completeness (%)	99.78	98.62
Redundancy	13.3	12.8
**Refinement**		
Resolution (Å)	2.89	3.18
No. reflections	108466	83898
R_{work} / R_{free}	0.201 / 0.230	0.172 / 0.234
Total number of atoms	30683	30673
Wilson B-factor (Å^2)	47.0	70.3
R.m.s deviations		
bond lengths (Å)	0.002	0.011
bond angles (°)	0.57	1.33
Average B factor (Å^2)		
Protein	40.49	67.85
Ligands	72.94	103.51
Ramachandran Plot		
outliers (%)	0.75	0.93
allowed (%)	3.92	6.57
favored (%)	95.33	92.50

Supplementary Table 2. Summary of cryo-EM data collection, processing and structure refinement.

	Human TNAP$^{18\text{-}500}$, in complex with scFv (EMDB-33865, PDB 7YIX)
**Data collection and processing**	
Magnification	105,000×
Voltage (kV)	300
Electron exposure (e-/Å^2)	50
Defocus range (μm)	-1.0 to -2.8
Pixel size (Å/pixel)	1.1
Symmetry imposed	C2
Initial particle images (no.)	10,037,359
Final particle images (no.)	314,858
Map resolution (Å)	2.96
FSC threshold	0.143
Map resolution range (Å)	2.8-4.6
**Refinement**	
Initial model used (PDB code)	7YIW
Model composition	
Non-hydrogen atoms	11080
Protein residues	1416
Ligands	14
B factors (Å^2)	
Protein	71.81
Ligand	81.68
R.m.s. deviations	
Bond length (Å)	0.003
Bond angles (°)	0.480
Validation	
MolProbity score	1.37
Clashscore	3.35
Rotamer outliers (%)	0.00
Ramachandran plot	
Favored (%)	96.37
Allowed (%)	3.63
Disallowed (%)	0.00

Supplementary References

1 Mornet, E. *et al.* Identification of fifteen novel mutations in the tissue-nonspecific alkaline phosphatase (TNSALP) gene in European patients with severe hypophosphatasia. *Eur J Hum Genet* **6**, 308-314, doi:10.1038/sj.ejhg.5200190 (1998).

2 Mumm, S. *et al.* Denaturing gradient gel electrophoresis analysis of the tissue nonspecific alkaline phosphatase isoenzyme gene in hypophosphatasia. *Mol Genet Metab* **75**, 143-153, doi:10.1006/mgme.2001.3283 (2002).

3 Mochizuki, H. *et al.* Severe hypercalcaemia and respiratory insufficiency associated with infantile hypophosphatasia caused by two novel mutations of the tissue-nonspecific alkaline phosphatase gene. *Eur J Pediatr* **159**, 375-379, doi:10.1007/s004310051290 (2000).

4 Taillandier, A. *et al.* Twelve novel mutations in the tissue-nonspecific alkaline phosphatase gene (ALPL) in patients with various forms of hypophosphatasia. *Hum Mutat* **18**, 83-84, doi:10.1002/humu.1154 (2001).

5 Spentchian, M. *et al.* Severe hypophosphatasia: characterization of fifteen novel mutations in the ALPL gene. *Hum Mutat* **22**, 105-106, doi:10.1002/humu.9159 (2003).

6 Mornet, E. *et al.* Hypophosphatasia: a genetic-based nosology and new insights in genotype-phenotype correlation. *Eur J Hum Genet* **29**, 289-299, doi:10.1038/s41431-020-00732-6 (2021).

7 Brun-Heath, I., Taillandier, A., Serre, J. L. & Mornet, E. Characterization of 11 novel mutations in the tissue non-specific alkaline phosphatase gene responsible for hypophosphatasia and genotype-phenotype correlations. *Mol Genet Metab* **84**, 273-277, doi:10.1016/j.ymgme.2004.11.003 (2005).

8 Sugimoto, N., Iwamoto, S., Hoshino, Y. & Kajii, E. A novel missense mutation of the tissue-nonspecific alkaline phosphatase gene detected in a patient with hypophosphatasia. *J Hum Genet* **43**, 160-164, doi:10.1007/s100380050061 (1998).

9 Del Angel, G., Reynders, J., Negron, C., Steinbrecher, T. & Mornet, E. Large-scale in vitro functional testing and novel variant scoring via protein modeling provide insights into alkaline phosphatase activity in hypophosphatasia. *Hum Mutat* **41**, 1250-1262, doi:10.1002/humu.24010 (2020).

10 Sanabria-de la Torre, R. *et al.* Characterization of Genetic Variants of Uncertain Significance for the ALPL Gene in Patients With Adult Hypophosphatasia. *Front Endocrinol (Lausanne)* **13**, 863940, doi:10.3389/fendo.2022.863940 (2022).

11 Taillandier, A. *et al.* Characterization of eleven novel mutations (M45L, R119H, 544delG, G145V, H154Y, C184Y, D289V, 862+5A, 1172delC, R411X, E459K) in the tissue-nonspecific alkaline phosphatase (TNSALP) gene in patients with severe hypophosphatasia. Mutations in brief no. 217. Online. *Hum Mutat* **13**, 171-172, doi:10.1002/(sici)1098-1004(1999)13:2<171::aid-humu16>3.0.co;2-t (1999).

12 Alonso, N. *et al.* Loss-of-Function Mutations in the ALPL Gene Presenting with Adult Onset Osteoporosis and Low Serum Concentrations of Total Alkaline Phosphatase. *J Bone Miner Res* **35**, 657-661, doi:10.1002/jbmr.3928 (2020).

13 Lia-Baldini, A. S. *et al.* A molecular approach to dominance in hypophosphatasia. *Hum Genet* **109**, 99-108, doi:10.1007/s004390100546 (2001).

14 Kato, M. *et al.* Novel mutation in the ALPL gene with a dominant negative effect in a Japanese family. *J Bone Miner Metab* **39**, 804-809, doi:10.1007/s00774-021-01219-0 (2021).

15 Braunstein, N. A. Multiple fractures, pain, and severe disability in a patient with adult-onset hypophosphatasia. *Bone Rep* **4**, 1-4, doi:10.1016/j.bonr.2015.10.005 (2016).

16 Whyte, M. P. *et al.* Hypophosphatasia: validation and expansion of the clinical nosology for children from 25 years experience with 173 pediatric patients. *Bone* **75**, 229-239, doi:10.1016/j.bone.2015.02.022 (2015).

17 Larsen, P. B., Skausig, O. B. & Jensen, E. A. Repeatedly low plasma alkaline phosphatase in a 56-year-old woman. A case of hypophosphatasia diagnosed in adulthood. *Pract Lab Med* **11**, 19-22, doi:10.1016/j.plabm.2018.02.001 (2018).

18 Fauvert, D. *et al.* Mild forms of hypophosphatasia mostly result from dominant negative effect of severe alleles or from

compound heterozygosity for severe and moderate alleles. *BMC Med Genet* **10**, 51, doi:10.1186/1471-2350-10-51 (2009).

19 Greenberg, C. R. *et al.* A homoallelic Gly317-->Asp mutation in ALPL causes the perinatal (lethal) form of hypophosphatasia in Canadian mennonites. *Genomics* **17**, 215-217, doi:10.1006/geno.1993.1305 (1993).

20 Hofmann, C. *et al.* Unexpected high intrafamilial phenotypic variability observed in hypophosphatasia. *Eur J Hum Genet* **22**, 1160-1164, doi:10.1038/ejhg.2014.10 (2014).

21 Henthorn, P. S., Raducha, M., Fedde, K. N., Lafferty, M. A. & Whyte, M. P. Different missense mutations at the tissue-nonspecific alkaline phosphatase gene locus in autosomal recessively inherited forms of mild and severe hypophosphatasia. *Proc Natl Acad Sci U S A* **89**, 9924-9928, doi:10.1073/pnas.89.20.9924 (1992).

22 Taillandier, A. *et al.* Fifteen new mutations (-195C>T, L-12X, 298-2A>G, T117N, A159T, R229S, 997+2T>A, E274X, A331T, H364R, D389G, 1256delC, R433H, N461I, C472S) in the tissue-nonspecific alkaline phosphatase (TNSALP) gene in patients with hypophosphatasia. *Hum Mutat* **15**, 293, doi:10.1002/(SICI)1098-1004(200003)15:3<293::AID-HUMU11>3.0.CO;2-Q (2000).

23 Brun-Heath, I. *et al.* Delayed transport of tissue-nonspecific alkaline phosphatase with missense mutations causing hypophosphatasia. *Eur J Med Genet* **50**, 367-378, doi:10.1016/j.ejmg.2007.06.005 (2007).

24 Reis, F. S., Gomes, D. C., Arantes, H. P. & Lazaretti-Castro, M. A two-year follow-up of asfotase alfa replacement in a patient with hypophosphatasia: clinical, biochemical, and radiological evaluation. *Arch Endocrinol Metab* **64**, 623-629, doi:10.20945/2359-3997000000222 (2021).

25 Saglam, H., Erdol, S. & Dorum, S. Clinical and Genetic Findings of Turkish Hypophosphatasia Cases. *J Clin Res Pediatr Endocrinol* **9**, 229-236, doi:10.4274/jcrpe.4549 (2017).

26 Orimo, H. *et al.* Mutational analysis and functional correlation with phenotype in German patients with childhood-type hypophosphatasia. *J Bone Miner Res* **16**, 2313-2319, doi:10.1359/jbmr.2001.16.12.2313 (2001).

27 Mao, X. *et al.* Two novel mutations in the ALPL gene of unrelated Chinese children with Hypophosphatasia: case reports and literature review. *BMC Pediatr* **19**, 456, doi:10.1186/s12887-019-1800-4 (2019).

28 Michigami, T. *et al.* Hypophosphatasia in Japan: ALPL Mutation Analysis in 98 Unrelated Patients. *Calcif Tissue Int* **106**, 221-231, doi:10.1007/s00223-019-00626-w (2020).

29 Maman, E., Briot, K. & Roux, C. Atypical femoral fracture in a 51-year-old woman: Revealing a hypophosphatasia. *Joint Bone Spine* **83**, 346-348, doi:10.1016/j.jbspin.2015.10.009 (2016).

30 Herasse, M. *et al.* Molecular study of three cases of odontohypophosphatasia resulting from heterozygosity for mutations in the tissue non-specific alkaline phosphatase gene. *J Med Genet* **40**, 605-609, doi:10.1136/jmg.40.8.605 (2003).

31 Watanabe, H., Hashimoto-Uoshima, M., Goseki-Sone, M., Orimo, H. & Ishikawa, I. A novel point mutation (C571T) in the tissue-non-specific alkaline phosphatase gene in a case of adult-type hypophosphatasia. *Oral Dis* **7**, 331-335, doi:10.1034/j.1601-0825.2001.00740.x (2001).

32 Daniel, A. B. *et al.* Healthcare resource utilization in the management of hypophosphatasia in three patients displaying a spectrum of manifestations. *Orphanet J Rare Dis* **13**, 142, doi:10.1186/s13023-018-0869-4 (2018).

33 Schmidt, T. *et al.* Clinical, radiographic and biochemical characteristics of adult hypophosphatasia. *Osteoporos Int* **28**, 2653-2662, doi:10.1007/s00198-017-4087-z (2017).

34 Yang, H. *et al.* Characterization of six missense mutations in the tissue-nonspecific alkaline phosphatase (TNSALP) gene in Chinese children with hypophosphatasia. *Cell Physiol Biochem* **32**, 635-644, doi:10.1159/000354467 (2013).

35 Goseki-Sone, M. *et al.* Hypophosphatasia: identification of five novel missense mutations (G507A, G705A, A748G, T1155C, G1320A) in the tissue-nonspecific alkaline phosphatase gene among Japanese patients. *Hum Mutat* **Suppl 1**, S263-267, doi:10.1002/humu.1380110184 (1998).

36 Zurutuza, L. *et al.* Correlations of genotype and phenotype in hypophosphatasia. *Hum Mol Genet* **8**, 1039-1046, doi:10.1093/hmg/8.6.1039 (1999).

37 Wenkert, D. *et al.* Hypophosphatasia: nonlethal disease despite skeletal presentation in utero (17 new cases and literature review). *J Bone Miner Res* **26**, 2389-2398, doi:10.1002/jbmr.454 (2011).

38 Makita, S. *et al.* A dimerization defect caused by a glycine substitution at position 420 by serine in tissue-nonspecific alkaline phosphatase associated with perinatal hypophosphatasia. *FEBS J* **279**, 4327-4337, doi:10.1111/febs.12022 (2012).

39 Michigami, T. *et al.* Common mutations F310L and T1559del in the tissue-nonspecific alkaline phosphatase gene are related to distinct phenotypes in Japanese patients with hypophosphatasia. *Eur J Pediatr* **164**, 277-282, doi:10.1007/s00431-004-1612-9 (2005).

40 Al-Shawafi, H. A., Komaru, K. & Oda, K. Molecular defect of tissue-nonspecific alkaline phosphatase bearing a substitution at position 426 associated with hypophosphatasia. *Mol Cell Biochem* **427**, 169-176, doi:10.1007/s11010-016-2908-6 (2017).

41 Ozono, K. *et al.* Identification of novel missense mutations (Phe310Leu and Gly439Arg) in a neonatal case of hypophosphatasia. *J Clin Endocrinol Metab* **81**, 4458-4461, doi:10.1210/jcem.81.12.8954059 (1996).

42 Chen, B. *et al.* A novel missense mutation in the ALPL gene causes dysfunction of the protein. *Mol Med Rep* **16**, 710-718, doi:10.3892/mmr.2017.6668 (2017).

43 Mornet, E. *et al.* Structural evidence for a functional role of human tissue nonspecific alkaline phosphatase in bone mineralization. *J Biol Chem* **276**, 31171-31178, doi:10.1074/jbc.M102788200 (2001).

44 Martins, L. *et al.* Novel ALPL genetic alteration associated with an odontohypophosphatasia phenotype. *Bone* **56**, 390-397, doi:10.1016/j.bone.2013.06.010 (2013).

45 Taillandier, A. *et al.* Genetic analysis of adults heterozygous for ALPL mutations. *J Bone Miner Metab* **36**, 723-733, doi:10.1007/s00774-017-0888-6 (2018).

46 Brun-Heath, I. *et al.* A case of lethal hypophosphatasia providing new insights into the perinatal benign form of hypophosphatasia and expression of the ALPL gene. *Clin Genet* **73**, 245-250, doi:10.1111/j.1399-0004.2007.00902.x (2008).

47 Nielson, C. M. *et al.* Rare coding variants in ALPL are associated with low serum alkaline phosphatase and low bone mineral density. *J Bone Miner Res* **27**, 93-103, doi:10.1002/jbmr.527 (2012).

48 Xu, L. *et al.* Four novel mutations in the ALPL gene in Chinese patients with odonto, childhood, and adult hypophosphatasia. *Biosci Rep* **38**, doi:10.1042/BSR20171377 (2018).

49 Castells, L., Cassanello, P., Muniz, F., de Castro, M. J. & Couce, M. L. Neonatal lethal hypophosphatasia: A case report and review of literature. *Medicine (Baltimore)* **97**, e13269, doi:10.1097/MD.0000000000013269 (2018).

50 Stevenson, D. A. *et al.* Autosomal recessive hypophosphatasia manifesting in utero with long bone deformity but showing spontaneous postnatal improvement. *J Clin Endocrinol Metab* **93**, 3443-3448, doi:10.1210/jc.2008-0318 (2008).

51 Reibel, A. *et al.* Orodental phenotype and genotype findings in all subtypes of hypophosphatasia. *Orphanet J Rare Dis* **4**, 6, doi:10.1186/1750-1172-4-6 (2009).

52 Ikenoue, S. *et al.* Discordant fetal phenotype of hypophosphatasia in two siblings. *Am J Med Genet A* **176**, 171-174, doi:10.1002/ajmg.a.38531 (2018).

53 Weiss, M. J. *et al.* A missense mutation in the human liver/bone/kidney alkaline phosphatase gene causing a lethal form of hypophosphatasia. *Proc Natl Acad Sci U S A* **85**, 7666-7669, doi:10.1073/pnas.85.20.7666 (1988).

54 Riancho-Zarrabeitia, L. *et al.* Clinical, biochemical and genetic spectrum of low alkaline phosphatase levels in adults. *Eur J Intern Med* **29**, 40-45, doi:10.1016/j.ejim.2015.12.019 (2016).

55 Oyachi, M. *et al.* A case of perinatal hypophosphatasia with a novel mutation in the ALPL gene: clinical course and review of the literature. *Clin Pediatr Endocrinol* **27**, 179-186, doi:10.1297/cpe.27.179 (2018).

56 Fedde, K. N., Michell, M. P., Henthorn, P. S. & Whyte, M. P. Aberrant properties of alkaline phosphatase in patient fibroblasts correlate with clinical expressivity in severe forms of hypophosphatasia. *J Clin Endocrinol Metab* **81**, 2587-2594, doi:10.1210/jcem.81.7.8675582 (1996).

57 Orimo, H. *et al.* Novel missense and frameshift mutations in the tissue-nonspecific alkaline phosphatase gene in a Japanese patient with hypophosphatasia. *Hum Mol Genet* **3**, 1683-1684, doi:10.1093/hmg/3.9.1683 (1994).

58 Utsch, B. *et al.* Infantile hypophosphatasia due to a new compound heterozygous TNSALP mutation - functional evidence for a hydrophobic side-chain? *Exp Clin Endocrinol Diabetes* **117**, 28-33, doi:10.1055/s-2008-1073157 (2009).

Supplementary Table 3. The list of primers.

TNAP-F	TTGGCGCGCCTTAGTGCCAGAGAAAGAGAAAGACCC
TNAP-R	CCGCGGCCGCGGCAGGAGCACAGTGGC
TNAP-A116T-F	CACCGCCACCACCTACCTGTGTGGGGTGAAG
TNAP-A116T-R	CACACAGGTAGGTGGTGGCGGTGCCGGCGC
TNAP-C201Y-F	GAGCCAGGGCTATAAGGACATCGCCTACCAG
TNAP-C201Y-R	CGATGTCCTTATAGCCCTGGCTCAAGGCCTC
TNAP-C489S-F	CGTATGCAGCCTCCATCGGGGCCAACCTCG
TNAP-C489S-R	GTTGGCCCCGATGGAGGCTGCATACGCCATC
TNAP-C497S-F	CCTCGGCCACTCTGCTCCTGCCGCGGCCGCTGAAAAC
TNAP-C497S-R	CGGCAGGAGCAGAGTGGCCGAGGTTGGCCCCGATG
TNAP-D156Y-F	GCTGGGCCAAGTACGCTGGGAAATCTGTGGGCATTG
TNAP-D156Y-R	GATTTCCCAGCGTACTTGGCCCAGCGCAGGATGGAG
TNAP-D214A-F	TGCATAACATCAGGGCCATTGACGTGATCATGGGGGGTG
TNAP-D214A-R	ATGATCACGTCAATGGCCCTGATGTTATGCATGAGCTGGTAG
TNAP-D25A-F	CAGAGAAAGAGAAAGCCCCCAAGTACTGGCGAGACCAAG
TNAP-D25A-R	CGCCAGTACTTGGGGGCTTTCTCTTTCTCTGGCACTAAG
TNAP-D294A-F	CGAGCCAGGGGCCATGCAGTACGAGCTGAACAG
TNAP-D294A-R	CTCGTACTGCATGCCCCCTGGCTCGAAGAGACC
TNAP-D306V-F	CAACGTGACGGTCCCGTCACTCTCCGAGATG
TNAP-D306V-R	GAGAGTGACGGGACCGTCACGTTGTTCCTGTTC
TNAP-D31A-F	CCAAGTACTGGCGAGCCCAAGCGCAAGAGACACTGAAATATG
TNAP-D31A-R	GTCTCTTGCGCTTGGGCTCGCCAGTACTTGGGGTCTTTC
TNAP-D370A-F	TGACCTCCTCGGAAGCCACTCTGACCGTGGTCACTG
TNAP-D370A-R	ACCACGGTCAGAGTGGCTTCCGAGGAGGTCAAGCTG
TNAP-D370E-F	CTCCTCGGAAGAGACTCTGACCGTGGTCAC
TNAP-D370E-R	CACGGTCAGAGTCTCTTCCGAGGAGGTCAAGC
TNAP-E235G-F	GAATAAAACTGATGTGGGGTATGAGAGTGACGAGAAAGCC
TNAP-E235G-R	GTCACTCTCATACCCCACATCAGTTTTATTCTTGGGGTAC
TNAP-E291K-F	CTATTGGGTCTCTTCAAGCCAGGGGACATGCAGTAC
TNAP-E291K-R	GTCCCCTGGCTTGAAGAGACCCAATAGGTAGTCC
TNAP-E311K-F	GACCCGTCACTCTCCAAGATGGTGGTGGTGGCCATC
TNAP-E311K-R	CCACCACCACCATCTTGGAGAGTGACGGGTCCGTCAC
TNAP-E35A-F	GAGACCAAGCGCAAGCGACACTGAAATATGCCCTGGAGC
TNAP-E35A-R	GCATATTTCAGTGTCGCTTGCGCTTGGTCTCGCCAGTACTTG
TNAP-E369A-F	GCTTGACCTCCTCGGCAGACACTCTGACCGTGGTCACT
TNAP-E369A-R	ACGGTCAGAGTGTCTGCCGAGGAGGTCAAGCTGCCTG

TNAP-G334D-R	GTGGTCAATTCTGTCTCCTTCCACCAGCAAGAAGAAGC
TNAP-G334R-F	GGTGGAAGGAGACAGAATTGACCACGGGCACCATG
TNAP-G420A-F	CAATGGGCCTGCCTACAAGGTGGTGGGCG
TNAP-G420A-R	CACCACCTTGTAGGCAGGCCCATTGCCATACAG
TNAP-G420S-F	GCAATGGGCCTAGCTACAAGGTGGTGGGCG
TNAP-G420S-R	CCACCTTGTAGCTAGGCCCATTGCCATACAGGATG
TNAP-G491R-F	GCAGCCTGCATCAGGGCCAACCTCGGCCAC
TNAP-G491R-R	CGAGGTTGGCCCTGATGCAGGCTGCATACGCCATC
TNAP-H171R-F	GAGAGTGAACCGTGCCACCCCCAGCGCCGC
TNAP-H171R-R	CTGGGGGTGGCACGGTTCACTCTCGTGGTGGTC
TNAP-H171Y-F	CGAGAGTGAACTATGCCACCCCCAGCGCCG
TNAP-H171Y-R	CTGGGGGTGGCATAGTTCACTCTCGTGGTGGTCAC
TNAP-H78A-F	CTCAAGGGTCAGCTCGCCCACAACCCTGGGGAGGAGAC
TNAP-H78A-R	TCCCCAGGGTTGTGGGCGAGCTGACCCTTGAGGATGC
TNAP-H79A-F	CAAGGGTCAGCTCCACGCCAACCCTGGGGAGGAGACCAG
TNAP-H79A-R	TCCTCCCCAGGGTTGGCGTGGAGCTGACCCTTGAGGATG
TNAP-I490F-F	GTATGCAGCCTGCTTCGGGGCCAACCTCGGC
TNAP-I490F-R	GAGGTTGGCCCCGAAGCAGGCTGCATACGCC
TNAP-K22A-F	GCCTTAGTGCCAGAGGCAGAGAAAGACCCCAAGTACTGGC
TNAP-K22A-R	TTGGGGTCTTTCTCTGCCTCTGGCACTAAGGCGCGCCC
TNAP-K264R-F	CAAACCGAGATACAGGCACTCCCACTTCATCTGGAAC
TNAP-K264R-R	GATGAAGTGGGAGTGCCTGTATCTCGGTTTGAAGCTCTTC
TNAP-K325A-F	CTGCGGAAGAACCCCGCAGGCTTCTTCTTGCTGGTGGAAG
TNAP-K325A-R	AGCAAGAAGAAGCCTGCGGGGTTCTTCCGCAGGATCTGGA
TNAP-K38A-F	GCGCAAGAGACACTGGCATATGCCCTGGAGCTTCAGAAGC
TNAP-K38A-R	AGCTCCAGGGCATATGCCAGTGTCTCTTGCGCTTGGTCTC
TNAP-K45A-F	GCCCTGGAGCTTCAGGCGCTCAACACCAACGTGGCTAAG
TNAP-K45A-R	CACGTTGGTGTTGAGCGCCTGAAGCTCCAGGGCATATTTCAG
TNAP-N170D-F	CCACGAGAGTGGACCATGCCACCCCCAGCG
TNAP-N170D-R	GGGTGGCATGGTCCACTCTCGTGGTGGTCACAATG
TNAP-N417S-F	CCTGTATGGCAGTGGGCCTGGCTACAAGGTG
TNAP-N417S-R	GTAGCCAGGCCCACTGCCATACAGGATGGC
TNAP-N430S-F	GTGAACGAGAGAGTGTCTCCATGGTGGACTATGC
TNAP-N430S-R	CACCATGGAGACACTCTCTCGTTCACCGCC
TNAP-N80A-F	GTCAGCTCCACCACGCCCCTGGGGAGGAGACCAGGCTG
TNAP-N80A-R	GTCTCCTCCCCAGGGGCGTGGTGGAGCTGACCCTTGAG

TNAP-P499N-F	GGCCACTGTGCTAATGCCGCGGCCGCTGAAAAC
TNAP-P499N-R	CGGCCGCGGCATTAGCACAGTGGCCGAGGTTG
TNAP-Q318A-F	GTGGTGGTGGCCATCGCGATCCTGCGGAAGAACCCCAAAG
TNAP-Q318A-R	TTCTTCCGCAGGATCGCGATGGCCACCACCACCATCTCGG
TNAP-R152H-F	CTCCATCCTGCACTGGGCCAAGGACGCTGGGAAATC
TNAP-R152H-R	GTCCTTGGCCCAGTGCAGGATGGAGGTGACCTCG
TNAP-R184W-F	CACTCGGCTGACTGGGACTGGTACTCAGACAAC
TNAP-R184W-R	GTACCAGTCCCAGTCAGCCGAGTGGGCGTAG
TNAP-R213A-F	CTCATGCATAACATCGCGGACATTGACGTGATCATGGGGGG
TNAP-R213A-R	GATCACGTCAATGTCCGCGATGTTATGCATGAGCTGGTAGG
TNAP-R321A-F	GCCATCCAGATCCTGGCGAAGAACCCCAAAGGCTTCTTC
TNAP-R321A-R	CCTTTGGGGTTCTTCGCCAGGATCTGGATGGCCACCAC
TNAP-R357L-F	GGTGGAGATGGACCTGGCCATCGGGCAGGCAGGCA
TNAP-R357L-R	CCTGCCCGATGGCCAGGTCCATCTCCACCGCCTCAT
TNAP-R391H-F	GATACACCCCCCATGGCAACTCTATCTTTGGTCTGGC
TNAP-R391H-R	GATAGAGTTGCCATGGGGGGTGTATCCACCAAATGTGAAG
TNAP-R450C-F	CTGTGCCCCTGTGCCACGAGACCCACGGCGG
TNAP-R450C-R	GGTCTCGTGGCACAGGGGCACAGCAGACTGC
TNAP-R71H-F	GACGGCTGCCCACATCCTCAAGGGTCAGC
TNAP-R71H-R	CCTTGAGGATGTGGGCAGCCGTCACTGTGGAG
TNAP-S364A-F	CGGGCAGGCAGGCGCCTTGACCTCCTCGGAAGACACT
TNAP-S364A-R	TCCGAGGAGGTCAAGGCGCCTGCCTGCCCGATGGCC
TNAP-S368A-F	GGCAGCTTGACCTCCGCGGAAGACACTCTGACCGTG
TNAP-S368A-R	GTCAGAGTGTCTTCCGCGGAGGTCAAGCTGCCTGCC
TNAP-S368del-F	CAGCTTGACCTCCGAAGACACTCTGACCGTGGTC
TNAP-S368del-R	GGTCAGAGTGTCTTCGGAGGTCAAGCTGCCTGC
TNAP-T167M-F	CATTGTGACCACCATGAGAGTGAACCATGCCACC
TNAP-T167M-R	GGTTCACTCTCATGGTGGTCACAATGCCCACAGATTTC
TNAP-T366N-F	CAGGCAGGCAGCTTGAACTCCTCGGAAGACACTCTGACCG
TNAP-T366N-R	GAGTGTCTTCCGAGGAGTTCAAGCTGCCTGCCTG
TNAP-Y263H-F	AGCTTCAAACCGAGACACAAGCACTCCCACTTCATCTGG
TNAP-Y263H-R	AGTGGGAGTGCTTGTGTCTCGGTTTGAAGCTCTTCCAGG
TNAP-Y28D-F	GAAAGACCCCAAGGACTGGCGAGACCAAGCGC
TNAP-Y28D-R	GGTCTCGCCAGTCCTTGGGGTCTTTCTCTTTCTCTG
T014130-F1	CATTGTCAGCTCCAGAGATGGAAC
T014130-R1	GACCTGAGCGTTGGTGTTATATG

T014130-F2	GACCTGAGCGTTGGTGTTATATG
T014130-R2	CATCTCCCAGGAACATGATGAC
