# Supplementary material for: The structural pathology for hypophosphatasia caused by malfunctional tissue non-specific alkaline phosphatase
Source: Nat Commun. 2023 Jul 8;14:4048. doi: 10.1038/s41467-023-39833-3 (PMC10329691; doi:10.1038/s41467-023-39833-3)
Supplement: Supplementary file 3 — Description of Additional Supplementary Files [file 41467_2023_39833_MOESM3_ESM.pdf]

File name: Supplementary Data 1

Description: Summary of TNAP disease-related mutations.
