# Supplementary material for: The structural pathology for hypophosphatasia caused by malfunctional tissue non-specific alkaline phosphatase
Source: Nat Commun. 2023 Jul 8;14:4048. doi: 10.1038/s41467-023-39833-3 (PMC10329691; doi:10.1038/s41467-023-39833-3)
Supplement: Supplementary file 5 — Reporting Summary [file 41467_2023_39833_MOESM5_ESM.pdf]

Corresponding author(s): Yu Cao
An Qin
Peixiang Ma

Last updated by author(s): Jun 13, 2023

Reporting Summary

Nature Portfolio wishes to improve the reproducibility of the work that we publish. This form provides structure for consistency and transparency in reporting. For further information on Nature Portfolio policies, see our Editorial Policies and the Editorial Policy Checklist.

Statistics

For all statistical analyses, confirm that the following items are present in the figure legend, table legend, main text, or Methods section.

n/a	Confirmed	
☐	☒	The exact sample size (*n*) for each experimental group/condition, given as a discrete number and unit of measurement
☐	☒	A statement on whether measurements were taken from distinct samples or whether the same sample was measured repeatedly
☐	☒	The statistical test(s) used AND whether they are one- or two-sided<br>*Only common tests should be described solely by name; describe more complex techniques in the Methods section.*
☒	☐	A description of all covariates tested
☒	☐	A description of any assumptions or corrections, such as tests of normality and adjustment for multiple comparisons
☐	☒	A full description of the statistical parameters including central tendency (e.g. means) or other basic estimates (e.g. regression coefficient) AND variation (e.g. standard deviation) or associated estimates of uncertainty (e.g. confidence intervals)
☐	☒	For null hypothesis testing, the test statistic (e.g. *F*, *t*, *r*) with confidence intervals, effect sizes, degrees of freedom and *P* value noted<br>*Give P values as exact values whenever suitable.*
☒	☐	For Bayesian analysis, information on the choice of priors and Markov chain Monte Carlo settings
☒	☐	For hierarchical and complex designs, identification of the appropriate level for tests and full reporting of outcomes
☒	☐	Estimates of effect sizes (e.g. Cohen's *d*, Pearson's *r*), indicating how they were calculated

Our web collection on statistics for biologists contains articles on many of the points above.

Software and code

Policy information about availability of computer code

Data collection	EPU software (version 2.12.0.2771REL)
Data analysis	MotionCorr2-1.1.0, RELION-3.1.1, cryoSPARC v3.2.2, UCSF Chimera v1.16, ChimeraX-1.4, COOT-0.8.9.2, Phenix v1.18.2, Refmac5 v7.1,pyem v0.56, Gautomatch v0.56, Flowjo v10, GraphPrism v9.0

For manuscripts utilizing custom algorithms or software that are central to the research but not yet described in published literature, software must be made available to editors and reviewers. We strongly encourage code deposition in a community repository (e.g. GitHub). See the Nature Portfolio guidelines for submitting code & software for further information.

Data

Policy information about availability of data

All manuscripts must include a data availability statement. This statement should provide the following information, where applicable:

- Accession codes, unique identifiers, or web links for publicly available datasets
- A description of any restrictions on data availability
- For clinical datasets or third party data, please ensure that the statement adheres to our policy

The coordinates are deposited at Protein Data Bank with accession code: 7YIV [https://www.rcsb.org/structure/7YIV], 7YIW [https://www.rcsb.org/structure/7YIW], and 7YIX [https://www.rcsb.org/structure/7YIX]. The cryo-EM maps have been deposited in the Electron Microscopy Data Bank (EMDB) with accession codes

EMD-33865 [https://www.ebi.ac.uk/emdb/search/EMD-33865]. The other relevant data generated in this study are provided in the Supplementary Information/ Source data file. Source data are provided with this paper.

Research involving human participants, their data, or biological material

Policy information about studies with human participants or human data. See also policy information about sex, gender (identity/presentation), and sexual orientation and race, ethnicity and racism.

Reporting on sex and gender	Female
Reporting on race, ethnicity, or other socially relevant groupings	Asian, Chinese
Population characteristics	3 female patients (age 50years, 58years and 61years) undergoing routine total hip replacement surgery at the Ninth People' s Hospital (Shanghai, China)
Recruitment	N/A
Ethics oversight	the Ethics Committee of the Ninth People's Hospital, Shanghai Jiaotong University School of Medicine (Ethics Number: SH9H-2022-TK36-1)

Note that full information on the approval of the study protocol must also be provided in the manuscript.

Field-specific reporting

Please select the one below that is the best fit for your research. If you are not sure, read the appropriate sections before making your selection.

☒ Life sciences ☐ Behavioural & social sciences ☐ Ecological, evolutionary & environmental sciences

For a reference copy of the document with all sections, see nature.com/documents/nr-reporting-summary-flat.pdf

Life sciences study design

All studies must disclose on these points even when the disclosure is negative.

Sample size	The structural data for biological macromolecules are validated by physical and chemical laws, instead of statistics. The biological sample size were indicated in the text.
Data exclusions	For structural experiments shown in the manuscript, all of the data were used. All the biochemical data were presented in the figure.
Replication	All the experiments have been repeated for at least three times, with a representative result shown in the figure. All the replication attempts are successful.
Randomization	For single particle analysis of EM, samples were allocated into experimental groups randomly. Randomization is not relevant to the flow cytometry experiments, as all data were used in a single analysis procedure.
Blinding	Blinding is not necessary or valid for the purposes of structure determination, flow cytometry.

Reporting for specific materials, systems and methods

We require information from authors about some types of materials, experimental systems and methods used in many studies. Here, indicate whether each material, system or method listed is relevant to your study. If you are not sure if a list item applies to your research, read the appropriate section before selecting a response.

Materials & experimental systems

n/a	Involved in the study	
☐	☒	Antibodies
☐	☒	Eukaryotic cell lines
☒	☐	Palaeontology and archaeology
☐	☒	Animals and other organisms
☒	☐	Clinical data
☒	☐	Dual use research of concern
☒	☐	Plants

Methods

n/a	Involved in the study	
☒	☐	ChIP-seq
☐	☒	Flow cytometry
☒	☐	MRI-based neuroimaging

Antibodies

Antibodies used	Mouse ALPL antibody (R&D Systems, Cat no: AF2910, Lot: WYM0221111, 1:200), beta actin Mouse monoclonal antibody (Affinity, Cat no: T0022, Lot: 54o2802, 1:1000), Anti-mouse IgG, HRP-linked Antibody (CST, Cat no: 7076P2, Lot: 36, 1:5000), Anti-rabbit IgG, HRP-linked Antibody (CST, Cat no: 7074P2, Lot: 25, 1:5000), Anti-mouse IgG (H+L) (DyLight™ 800 4X PEG Conjugate) (CST, Cat no: 5257, Lot: 11, 1:5000), Rabbit Anti-Goat IgG H&L (HRP) (Abcam, Cat no: ab6741, Lot: GR3324245-8, 1:5000) , HRP-conjugated DYKDDDDK Tag Monoclonal antibody (ProteinTech, Cat no : HRP-66008, Lot: 21006202, 1:10000), anti-human TNAP antibody developed in this manuscript (JTALP001),
Validation	anti-human TNAP JTALP001 was validated as shown in Method and supplementary figure 1e.<br>Validation statement available at the web page for each antibody:<br>Mouse ALPL antibody: https://www.rndsystems.com/cn/products/mouse-alkaline-phosphatase-alpl-antibody_af2910#product-citations<br>beta actin Mouse monoclonal antibody: https://www.affbiotech.cn/goods-6281-T0022-beta_Actin_Antibody.html<br>HRP-conjugated DYKDDDDK Tag Monoclonal antibody: https://www.ptglab.co.jp/Products/Flag-Tag-Antibody-HRP-66008.htm

Eukaryotic cell lines

Policy information about cell lines and Sex and Gender in Research

Cell line source(s)	Hi5 cell line (Thermo Fisher Scientific, B85502) ; Expi293F cell line (Thermo Fisher Scientific, A14527); HEK293T cell line (NCACC, SCSP-502); MC3T3-E1 cell line (ATCC, CRL-2593)
Authentication	The Hi5, Expi293F, HEK293T cell lines are used to produce proteins for structure determination and analysis. They were purchased and the authentication are not conducted. MC3T3-E1 cell line was purchased from National Collection of Authenticated Cell Cultures and it was validated by STR profiling.
Mycoplasma contamination	No mycoplasma contamination was detected.
Commonly misidentified lines<br>(See ICLAC register)	To our best knowledge, there is no commonly misidentified lines.

Animals and other research organisms

Policy information about studies involving animals; ARRIVE guidelines recommended for reporting animal research, and Sex and Gender in Research

Laboratory animals	Alpl+/– mice and wild type mice were maintained on C57BL/6J background. Mouse pre-osteoblasts were harvested from murine calvarial bones of 1-week-old C57BL/6J mice.
Wild animals	The study did not involve wild animals.
Reporting on sex	All mice were used for analysis regardless of sex.
Field-collected samples	The study did not involve samples collected from the field.
Ethics oversight	All of the procedures that involved animals were approved by the Institutional Animal Care and Ethics Committee of the Ninth People's Hospital, Shanghai Jiaotong University School of Medicine (Shanghai, China; Ethics Number: SH9H-2022-A037-SB).

Note that full information on the approval of the study protocol must also be provided in the manuscript.

Flow Cytometry

Plots

Confirm that:

- [x] The axis labels state the marker and fluorochrome used (e.g. CD4-FITC).
- [x] The axis scales are clearly visible. Include numbers along axes only for bottom left plot of group (a 'group' is an analysis of identical markers).
- [x] All plots are contour plots with outliers or pseudocolor plots.
- [x] A numerical value for number of cells or percentage (with statistics) is provided.

Methodology

Sample preparation	Cells were collected and re-suspended in an ice-cold FACS buffer, containing PBS, 0.05 % BSA and 2 mM EDTA. The TNAP protein expressing cells were then incubated with primary antibody for 20 min on ice, and washed with 1 mL ice-cold FACS buffer, spun, and re-suspended in a 100 μL ice-cold FACS buffer containing the secondary antibody. After incubating on ice

for 15 min, the cells were washed twice and re-suspended in a FACS buffer. The harvested cells were sorted and analyzed on a flow cytometer.

Instrument	CytoFLEX S, Beckman Culter
Software	FlowJo, V10
Cell population abundance	The population abundance was provided in supplementary Figure 11.
Gating strategy	HEK293T cells was used to set the gates. HEK293T cells transfected with EGFP was used as negative control. The gating strategy was shown in supplementary Figure 11.

☒ Tick this box to confirm that a figure exemplifying the gating strategy is provided in the Supplementary Information.
